# A novel mutation in the glycogen synthase 2 gene in a child with glycogen storage disease type 0

**DOI:** 10.1186/1471-2350-11-3

**Published:** 2010-01-05

**Authors:** Ana Priscila Soggia, Maria Lúcia Correa-Giannella, Maria Angela Henriques Fortes, Ana Mercedes Cavaleiro Luna, Maria Adelaide Albergaria Pereira

**Affiliations:** 1 Divisão de Endocrinologia do Hospital das Clínicas da Faculdade de Medicina da Universidade de São Paulo, Dr Eneas de Carvalho Aguiar Street, 647, 05403-000, São Paulo, Brazil; 2 Laboratório de Endocrinologia Celular e Molecular LIM-25, Faculdade de Medicina da Universidade de São Paulo, Dr Arnaldo Street, 455, room 4305, São Paulo, Brasil

## Abstract

**Background:**

Glycogen storage disease type 0 is an autosomal recessive disease presenting in infancy or early childhood and characterized by ketotic hypoglycemia after prolonged fasting and postprandial hyperglycemia and hyperlactatemia. Sixteen different mutations have been identified to date in the gene which encodes hepatic glycogen synthase, resulting in reduction of glycogen storage in the liver.

**Case Presentation:**

Biochemical evaluation as well as direct sequencing of exons and exon-intron boundary regions of the *GYS2 *gene were performed in a patient presenting fasting hypoglycemia and postprandial hyperglycemia and her parents. The patient was found to be compound heterozygous for one previously reported nonsense mutation (c.736 C>T; R243X) and a novel frameshift mutation (966_967delGA/insC) which introduces a stop codon 21 aminoacids downstream from the site of the mutation that presumably leads to loss of 51% of the COOH-terminal part of the protein. The glycemia and lactatemia of the parents after an oral glucose tolerance test were evaluated to investigate a possible impact of the carrier status on the metabolic profile. The mother, who presented a positive family history of type 2 diabetes, was classified as glucose intolerant and the father, who did not exhibit metabolic changes after the glucose overload, had an antecedent history of hypoglycemia after moderate alcohol ingestion.

**Conclusion:**

The current results expand the spectrum of known mutations in *GYS2 *and suggest that haploinsufficiency could explain metabolic abnormalities in heterozygous carriers in presence of predisposing conditions.

## Background

Glycogen storage disease type 0 (GSD0) is an autosomal recessive disease presenting in infancy or early childhood caused by mutations in the *GYS2 *[1], a gene located on chromosome 12p 12.2 composed of 16 exons which codes for the hepatic isoform of glycogen synthase (GS) [2]. This rate-limiting enzyme of 80.9 kDa participates in the production of glycogen, the glucose polymer which constitutes the major repository of readily available energy [3-5].

Glucose use is strictly controlled and abnormal glucose handling is associated with some human diseases, such as glycogen storage diseases [3] and diabetes [6]. GS is a key component of the hepatic synthesis of glycogen, catalyzing the successive addition of α-1,4-linked glucose residues to the nonreducing end of glycogen [4]. Impaired activity of GS results in great reduction of glycogen storage in the liver, which renders the patient prone to develop ketotic hypoglycemia and low lactatemia after prolonged fasting. The inability to synthesize hepatic glycogen shifts meal-derived glucose from glycogenesis to the glycolytic pathway and explains the findings of hyperglycemia, hyperlactatemia and hyperlipidemia in the postprandial period [7-9].

The current gold standard for diagnosis is molecular analysis of the affected gene, which replaced liver biopsy [7]. Sixteen different mutations have been identified to date [1,7,8,10]. In the present report, we describe a novel deletion insertion mutation in the *GYS2 *gene in a Brazilian child as well as the glycemia and lactatemia of her parents after an oral glucose tolerance test, to investigate a possible impact of the carrier status on the metabolic response to glucose overload.

## Case Presentation

This third female child of healthy nonconsanguineous parents was born at full term without complications weighting 3.050 g and had normal psychomotor development. At 6 months of age she developed sweating during fast, mainly in the mornings. At 2 years measurements of capillary glycemia revealed fasting hypoglycemia (1.94 mmol/L) with ketonemia and postprandial hyperglycemia (11.3 mmol/L). At 6 years of age, the patient was evaluated at the Endocrinology Department of Hospital das Clinicas for diagnosis confirmation. Her physical examination was normal, without hepatomegaly or neurological abnormalities. Her weight and height were in the 25^th ^and 10-25^th ^percentiles, respectively. Her HbA1c was 5.2%.

None of the patient's first-degree relatives had a positive history of hypoglycemic episodes during fasting however the father presented some episodes of hypoglycemia after moderate alcohol ingestion (5 to 7 ethanol doses, approximately 90 grams of ethanol) during adolescence. In two occasions, he was taken to the hospital and had documented hypoglycemia (capillary blood glucose concentrations between 2.5 and 2.7 mmol/L) and improvement of the symptoms after glucose infusion. The mother presented a positive family history of type 2 diabetes, a BMI of 24.3 kg/m^2^, an HbA1c of 6.3%, a basal insulinemia of 5.6 μU/mL and a Homeostatic Model Assessment (HOMA)-IR of 1.08.

The biochemical investigation was carried out as follows: hourly measurements of plasma glucose, ketones, insulin, C-peptide and blood lactate concentrations were performed during fasting. After the plasma glucose concentration decreased to < 2.7 mmol/L, glucagon (0.03 mg/kg) was administered intravenously, and plasma glucose and blood lactate concentrations were measured at 30 and 60 minutes. Thereafter, the patient had a mixed meal with measurements of plasma glucose and blood lactate every 30 minutes for 3 hours as previously described by Bachrach and Weinstein [7,8]

Cortisol, insulin, C-peptide were measured by a immunofluorimetric assay (Wallac AutoDELFIA, Turku, Finland), ACTH and IGF-1 were measured by a immunometric assay (DPC Immulite, Los Angeles, USA). The plasma glucose and blood lactate were measured by the enzymatic method.

Following written consent, which was obtained from the patient's parents for publication of study, blood samples were collected from the proband and her parents for mutation screening. Polymerase chain reaction (PCR) and direct sequencing of exons and exon-intron boundary regions of the *GYS2 *gene was performed as previously described [1] in genomic DNA.

Plasma glucose and blood lactate concentrations were obtained from the parents before and every 30 minutes for 2 hours after a 75-g oral glucose tolerance test (OGTT). The study was carried out in compliance to the Institution's Ethics Committee and in accordance to The Declaration of Helsinki, with informed and free consent being required of each subject or subject's guardian.

**Figure 1:**
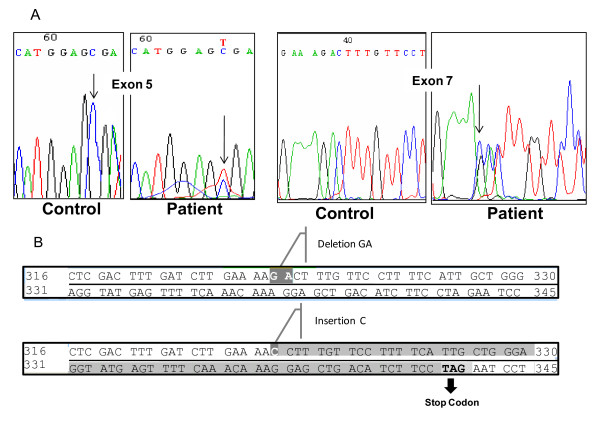
**Sequencing electropherograms showing *GYS2 *mutations**. **A: **Sequencing electropherograms showing the substitution of C to T at nucleotide 736 resulting in a stop codon at exon 5 (c.736 C>T; R243X) (Upper panel) and the exon 7 deletion (GA) plus insertion (C) mutation at nucleotides 966-967 (966_967delGA/insC) also resulting in a premature stop codon (bottom panel). **B**: Frameshift in the codon sequence which is predicted to result in truncation of the GYS2 protein at amino acid 343.

Ketotic hypoinsulinemic hypoglycemia and absence of hormonal deficiencies were demonstrated after a 5-hour fast (Table 1). Glucagon infusion did not promote a normal glycemic response, and a mixed meal resulted in postprandial hyperglycemia and hyperlactatemia (Table 2). Direct sequencing of the coding region of the *GYS2 *gene revealed that the affect child was compound heterozygous for two mutations: a novel mutation resulting from deletion/insertion (966_967delGA/insC at codons 322/323 in exon 7) resulting in overlapping peaks beginning at nucleotide c.966 (filled arrow) and a previously described nonsense mutation which causes the substitution of an arginine (CGA) for a stop codon (TGA) (c.736 C>T at codon 246 in exon 5; R246X) [1] (Figure 1). The 966_967delGA/insC mutation results in a frameshift of the reading frame from codon 323, leading to a premature stop codon (codon 343). Genetic analysis of the parents demonstrated that the mother was carrier of the mutation c.736 C>T and the father was carrier of the mutation 966_967 delGAinsC (Table 3).

**Table 1:** Metabolic and hormonal response to fasting (5 hours)

***Glycemia ***(3,33-6,05 mmol/L)	1,16
***Insulinemia ***(< 173 pmol/L)	**< 15**

***C-Peptide ***(0.13-1,2 nmol/L)	**< 0.15**

***Ketonemia ***(< 0.5 mmol/L)	**4.7**

*IGF-1 *(2,62-22,3 nmol/L)	4,45

*ACTH *(<13,2 pmol/L)	7,48

*Cortisol *(193-855 nmol/L)	549

**Table 2:** Response of plasma glucose, lactate and insulin to fasting, glucagon challenge and to carbohydrate containing-meal

***Time*** ***(min)***	***Glucose*** ***(mmol/L)***	***Lactate*** ***(0,49-1,98 mmol/L)***	***Insulin*** *(pmol/L)*
***After 5 h Fast***			

***0***	3,21	3,77	-

***60***	2,66	1,99	-

***150***	2,27	1,55	-

***300***	1,98	1,33	< 17

***361***	***Glucagon Infusion EV***		

***390***	3,13	1,76	-

***420***	1,83	1,65	< 17

***Mixed Meal***			

***60***	11,32	5,77	316,4

***90***	9,26	6,54	332,4

***120***	10,21	7,54	259,5

***150***	7,1	6,98	197,8

**Table 3:** First-degree relatives' genetic analysis and response of plasma glucose and lactate to fasting and oGTT

		Basal*	*30 min*	*60 min*	*120 min*	*Genetic*
Mother	*Glycemia*	4,88	7,82	9,43	**7,99**	Heterozygote for c.736 C >T (R246X)
(31 years)	*Lactate*	0,44	0,75	1,15	1,06	
	*Ketonemia*	0,8				

Father	*Glycemia*	4,71	7,15	5,88	4,21	Heterozygote for 966_967 delGAinsC
(33 years)	*Lactate*	0,65	1,64	0,93	1,95	
	*Ketonemia*	1,2				

* 12-h fasting; oGTT: oral glucose tolerance test with 75 g glucose

As shown on Table 3, the parents did not present hypoglycemia after a 12-hour fasting period and the mother was classified as glucose intolerant.

Patient was recommended to start a diet enriched in proteins and in low glycemic carbohydrates, especially cornstarch (1-1.5 g/kg), after which the hypoglycemic episodes became less frequent and less intense.

The present study reports the identification of a novel deletion/insertion mutation in the *GYS2 *gene in a patient with GSD0. As far as we know, this is the 17^th ^different mutation described thus far [7,8,10], the first in a Brazilian patient. The mutations T445M in exon 11 [7,10] and R246X in exon 5 [1,8] were found in more than one family, including the family reported here, which has European ancestry and harbors the latter nonsense mutation. On the second allele, our patient was found to have a new frameshift mutation comprised of a 2 bp deletion and a 1 bp insertion which introduced a stop codon 21 aminoacids downstream from the site of the mutation (aminoacid 343) that presumably leads to loss of 51% of the COOH-terminal part of the protein, including potential phosphorylation sites as well as a highly conserved region believed to contain the glucose-6-P binding site (aminoacids 501-600) [11]. This predicted loss could result in impaired or null GS activity, which is known to be regulated by binding allosteric ligands, most notably glucose-6-P, and by covalent phosphorylation [3].

The mutations described so far seem to point out the absence of genotype-phenotype correlation, as exemplified by mutation R246X, which leads to loss of 65% of the COOH-terminal part of the protein and total lack of GS activity demonstrated by a functional study [1]. Orho et al reported a homozygous patient for this mutation presenting practically normal hepatic glycogen. Besides, absence of fasting hypoglycemia and postprandial hyperglycemia were documented in heterozygous carriers of this mutation [1]. In the presently studied family, the mother was carrier of the R246X mutation and did not develop fasting hypoglycemia however she presented glucose intolerance in the OGTT. Based on the finding of fasting hypoglycemia in one heterozygous carrier of a mutation in the 5'-donor splicing site of intron 6, that predictably results in skipping of exon 6 and a premature stop codon in exon 7, Orho et al. [1] proposed that this truncated protein might disturb the structure of the wild-type GS, which is believed to be a dimer [3] acting in a dominant-negative manner.

An alternative possibility for the finding of metabolic abnormalities in some heterozygous carriers could be haploinsufficiency in the presence of other predisposing conditions, in a mechanism similar to the one proposed to explain how abnormal gene dosage is associated with human birth defects: increased sensitivity to effects of environmental insults [12]. In the family presented herein, both parents presented predisposing conditions to metabolic abnormalities: the mother, classified as glucose intolerant, have a positive family history of type 2 diabetes and the father presented episodes of hypoglycemia after moderate alcohol ingestion. Alcohol is known to inhibit gluconeogenesis and is therefore more likely to contribute to the development of hypoglycemia when glycogen stores are low [13]. The absence of hyperlactatemia after glucose overload in both parents could speak against the hypothesis of haploinsufficiency however this biochemical feature may be absent even in patients with GSD0 [7].

The present data are insufficient to draw any conclusion regarding the susceptibility of heterozygous carriers of *GYS2 *mutations to glucose metabolism abnormalities, especially because a previous linkage study did not identify this as a major gene contributing to type 2 diabetes susceptibility [14]. However, they may suggest that in presence of predisposing conditions, carriers of *GYS2 *mutations might be at increased risk of developing hypoglycemia.

## Conclusions

The current results expand the spectrum of known mutations in *GYS2 *and suggest that haploinsufficiency could explain metabolic abnormalities in heterozygous carriers in presence of predisposing conditions.

## Abbreviations

(GSD0): Glycogen storage disease type 0; (GS): glycogen synthase; (PCR): polymerase chain reaction; (OGTT): oral glucose tolerance test.

## Competing interests

The authors declare that they have no competing interests.

## Authors' contributions

APS and MAAP diagnosed the patient, interpreted the clinical results, and participated in the editing of the manuscript. MLCG interpreted the molecular results, and wrote the draft of the paper. MAHF and AMCL carried out the molecular genetic studies, and were involved in the writing of the manuscript. All authors have read and approved the final manuscript.

## Pre-publication history

The pre-publication history for this paper can be accessed here:

http://www.biomedcentral.com/1471-2350/11/3/prepub
